# Atraumatic Splenic Rupture as the Initial Manifestation of Splenic Marginal Zone Lymphoma: A Case Report

**DOI:** 10.7759/cureus.100773

**Published:** 2026-01-04

**Authors:** Rodrigo Furlan Silva Fabri, Anvitha Soundararajan, Rodolfo Myronn de Melo Rodrigues, Gowri Renganathan, M Nawar Hakim, Uttamkumar Savariya, Lela Ruck

**Affiliations:** 1 Internal Medicine, Texas Tech University Health Sciences Center El Paso Paul L. Foster School of Medicine, El Paso, USA; 2 Pathology, Texas Tech University Health Sciences Center El Paso Paul L. Foster School of Medicine, El Paso, USA

**Keywords:** atraumatic splenic rupture, clinical case report, non-hodgkin lymphoma (nhl), post-splenectomy, splenic marginal zone lymphoma, spontaneous hemoperitoneum

## Abstract

Atraumatic splenic rupture (ASR) is a rare but life-threatening emergency that is often linked to previously unrecognized systemic disease, including hematologic malignancies. We report the case of a middle-aged man who presented with sudden abdominal pain and hemodynamic instability and was found to have spontaneous splenic rupture requiring urgent hemodynamic stabilization, splenic artery embolization, and splenectomy. Histopathologic evaluation of the resected spleen revealed an underlying splenic marginal zone lymphoma (SMZL) that had not been clinically apparent before the acute event. The patient recovered well postoperatively with appropriate vaccinations and hematology follow-up. This case highlights the need to consider occult lymphoproliferative disorders in patients with spontaneous splenic rupture and underscores the importance of prompt multidisciplinary management to prevent catastrophic hemorrhage and secure a definitive diagnosis.

## Introduction

Atraumatic splenic rupture (ASR) is an uncommon but potentially life-threatening clinical emergency defined as splenic disruption occurring in the absence of antecedent trauma. Although it represents less than 1% of all splenic ruptures, the associated mortality is markedly higher due to delays in recognition and the frequency of rapid hemodynamic deterioration [1,2]. Numerous underlying conditions have been implicated in atraumatic rupture, including infectious processes, inflammatory disorders, and medication-related effects. Among these, hematologic malignancies, particularly lymphomas and leukemias, account for a significant proportion of reported cases and are recognized as major predisposing factors for severe intra-abdominal hemorrhage [3-5].

Splenic marginal zone lymphoma (SMZL) is a rare, indolent B-cell non-Hodgkin lymphoma characterized by progressive splenomegaly, bone marrow involvement, and occasional circulating villous lymphocytes [6]. Most patients are asymptomatic at presentation, and the disease is often detected incidentally through imaging or hematologic evaluation. Despite the spleen being the primary site of involvement, spontaneous rupture is exceedingly uncommon and has been documented primarily through isolated case reports and small case series [7]. When it occurs, rupture represents a rare but serious complication with substantial diagnostic and therapeutic implications.

The mechanisms proposed to contribute to splenic rupture in this lymphoma include progressive lymphoid infiltration of the white pulp, rising intrasplenic pressure, capsular attenuation, and ischemic injury, pathological changes that weaken the splenic architecture and predispose the organ to spontaneous tearing [3,5]. Because symptoms may be nonspecific and clinical deterioration may occur abruptly, prompt imaging with contrast-enhanced computed tomography (CT) is essential for early identification and appropriate management.

Given the rarity of atraumatic splenic rupture and its strong association with underlying pathology, clinicians must maintain a broad differential diagnosis and consider occult hematologic malignancy when evaluating cases of spontaneous splenic disruption. Awareness of this relationship is critical to facilitating timely diagnosis and guiding urgent therapeutic decision-making.

## Case presentation

A 54-year-old Hispanic man with no known medical history presented to the emergency department with five days of progressively worsening right upper quadrant abdominal pain radiating to the left flank. The pain was sharp, constant, and associated with intermittent chills and subjective fever. He denied recent trauma, infection, anticoagulant use, or strenuous activity. There were no constitutional symptoms such as weight loss or night sweats.

On arrival, he appeared acutely ill and diaphoretic. His blood pressure was 89/59 mmHg, and his heart rate was 160 beats per minute. The abdomen was distended with diffuse tenderness, most prominent in the left upper quadrant, with guarding and rebound. Laboratory studies revealed a hemoglobin of 9.5 g/dL, leukocytosis of 12.4 × 10⁹/L, and a serum creatinine of 2 mg/dL.

**Figure 1:**
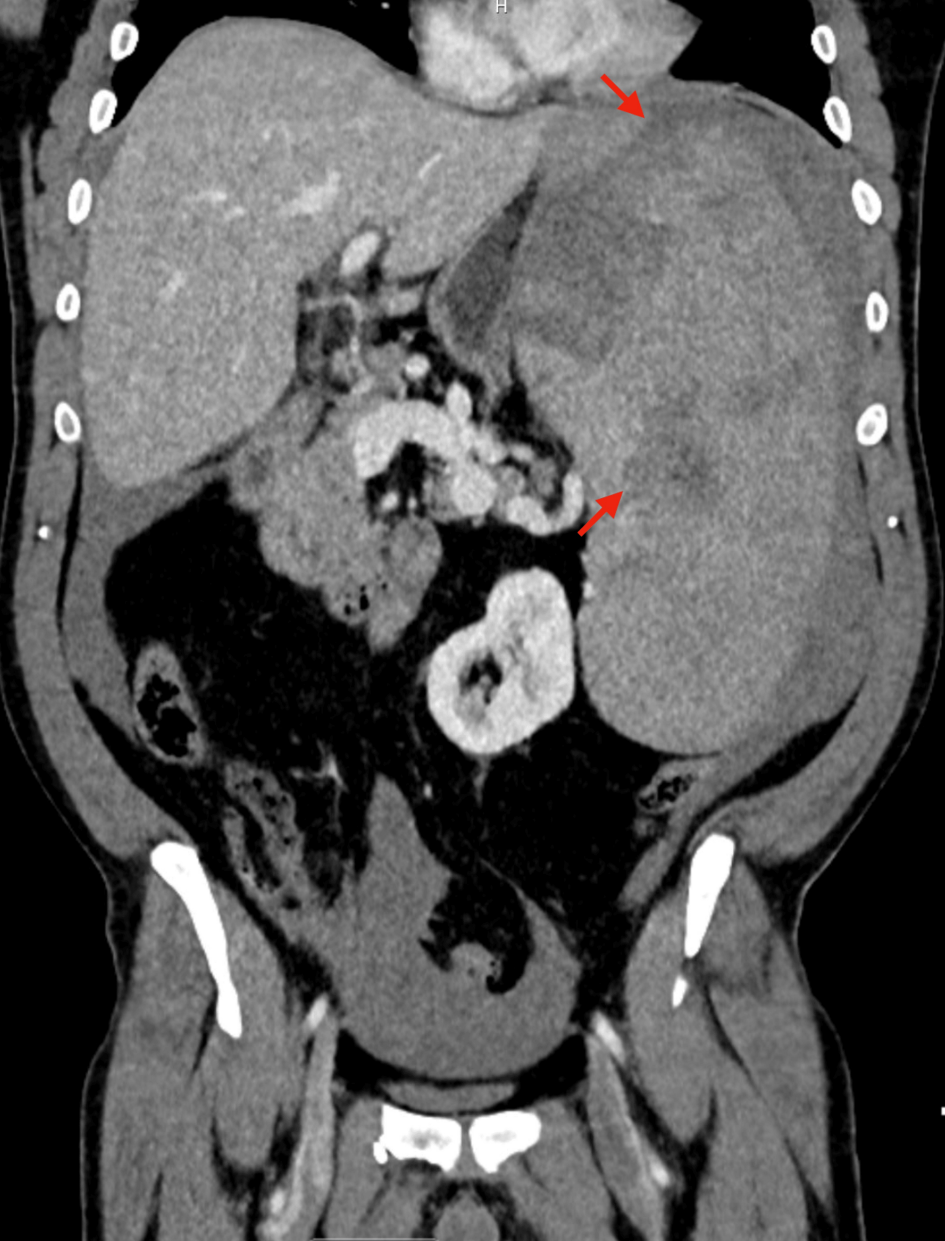
Coronal contrast-enhanced CT demonstrating atraumatic splenic rupture Coronal contrast-enhanced CT of the abdomen showing marked splenomegaly with multifocal parenchymal lacerations (red arrows) and large-volume hemoperitoneum. CT: computed tomography

**Figure 2:**
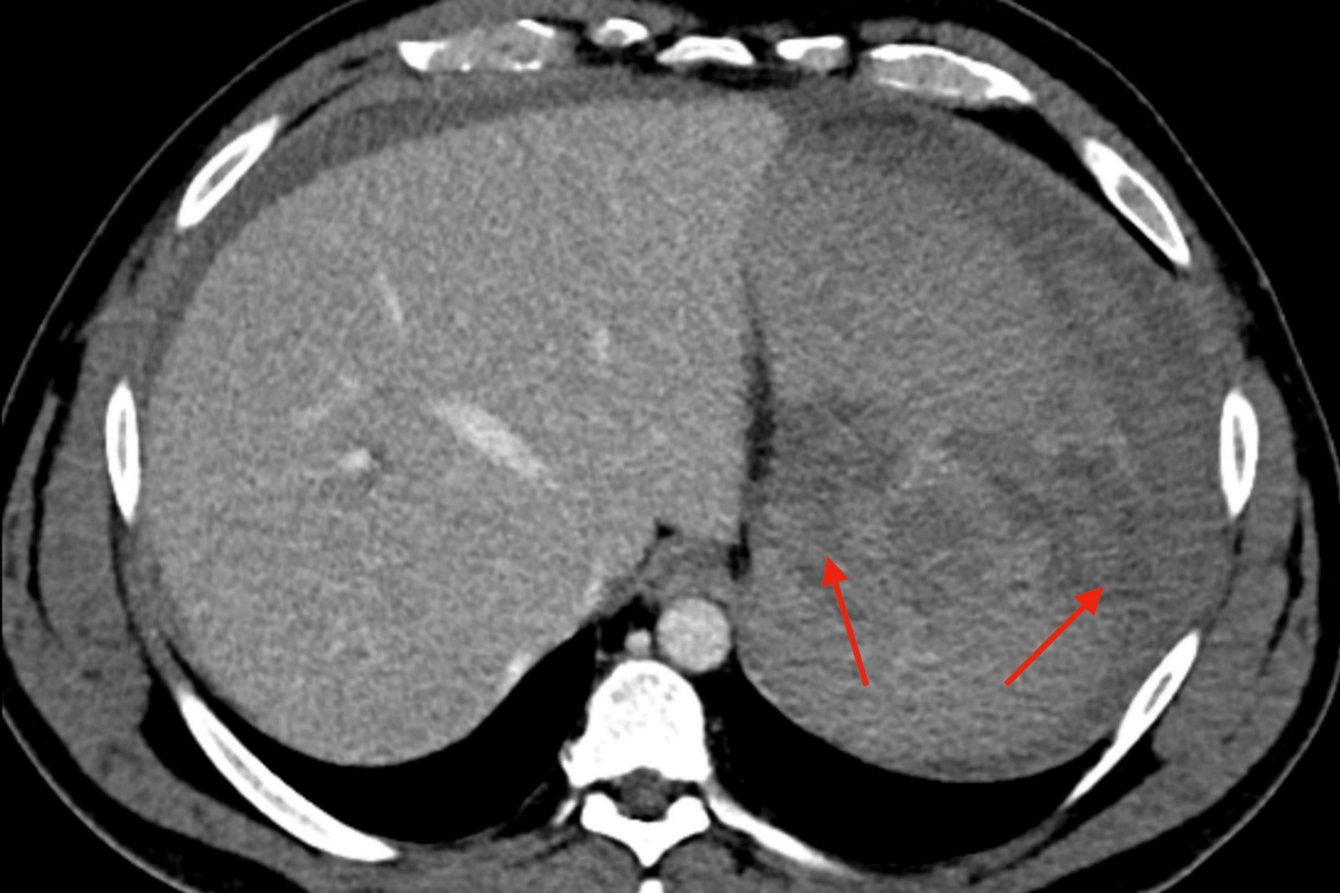
Axial contrast-enhanced CT findings of atraumatic splenic rupture Axial contrast-enhanced CT of the abdomen demonstrating irregular splenic contour with deep parenchymal lacerations (red arrows) and surrounding high-attenuation hemoperitoneum. CT: computed tomography

Contrast-enhanced computed tomography (CT) of the abdomen and pelvis demonstrated massive splenomegaly with multifocal parenchymal lacerations and a large volume of hemoperitoneum (Figure 1 and Figure 2). No evidence of hepatic injury, bowel perforation, or other visceral trauma was seen.

The patient received aggressive resuscitation with intravenous crystalloids and packed red blood cells. Due to persistent hemodynamic instability, emergent splenic artery embolization was performed. Although bleeding initially decreased, he developed recurrent hypotension within hours, prompting emergency exploratory laparotomy. A total splenectomy was performed, evacuating approximately 2.7 liters of intraperitoneal blood.

**Figure 3:**
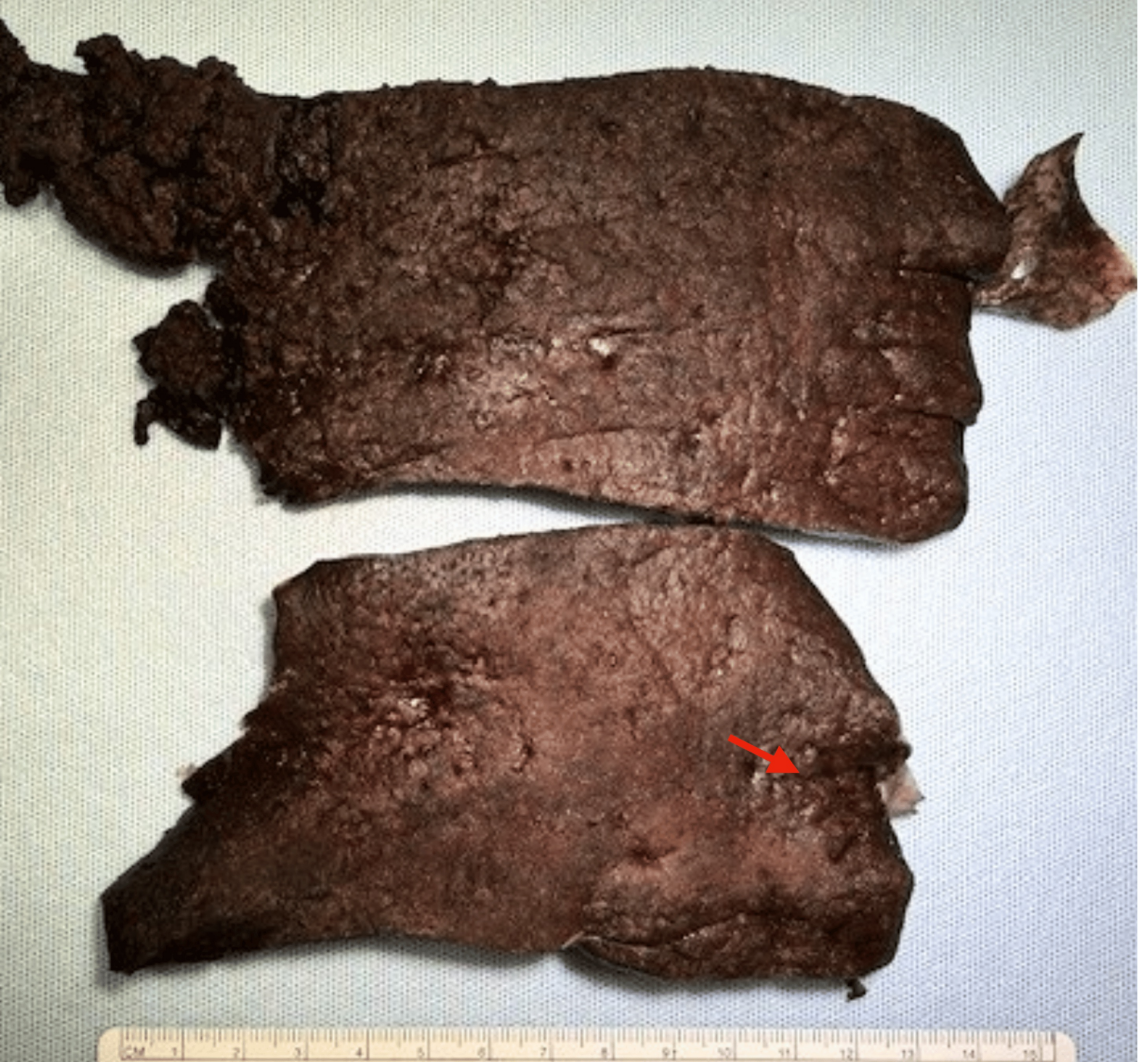
Gross pathological features of the spleen Gross splenic specimen demonstrating extensive splenomegaly with diffuse capsular thinning and large subcapsular hematomas (red arrow). The markedly enlarged spleen shows near-complete replacement of the normal parenchyma by marginal zone lymphoma, contributing to capsular fragility and predisposition to atraumatic rupture.

Gross pathological examination revealed a markedly enlarged spleen weighing 5,118 g (Figure 3) with diffuse capsular thinning and extensive subcapsular hematomas.

**Figure 4:**
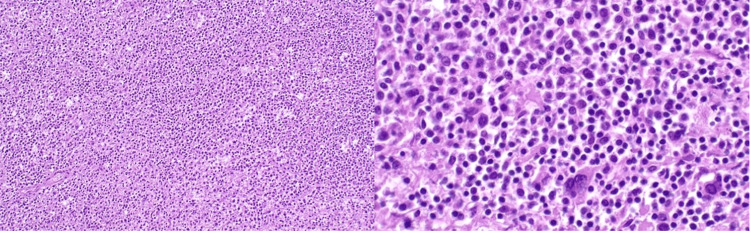
Histopathologic features of splenic marginal zone lymphoma H&E-stained sections of the spleen showing complete effacement of normal splenic architecture by a monotonous infiltrate of lymphoma cells. Low-power view at 100× magnification (left) demonstrates expansion of the white pulp and diffuse replacement of the parenchyma. High-power view at 400× magnification (right) reveals atypical small-to-medium-sized B lymphocytes with irregular nuclear contours, consistent with marginal zone lymphoma. H&E: hematoxylin and eosin

Microscopic evaluation demonstrated expansion of the white pulp by small-to-medium atypical lymphocytes with effacement of normal follicular architecture. Hematoxylin and eosin (H&E) staining highlighted sheets of neoplastic marginal zone-type B cells (Figure 4).

Immunohistochemical analysis showed strong membranous positivity for CD20, CD79a, and BCL2, with negative staining for CD10, CD23, and cyclin D1. The Ki-67 proliferation index was approximately 5%. These findings were diagnostic of splenic marginal zone lymphoma.

Postoperatively, the patient stabilized rapidly. He tolerated diet advancement by postoperative day 3 and was discharged on day 5. Prior to discharge, he received guideline-recommended vaccinations against *Streptococcus pneumoniae*, *Neisseria meningitidis*, and *Haemophilus influenzae* type b. He was referred to hematology-oncology for further staging and long-term management.

## Discussion

Atraumatic splenic rupture (ASR) is an uncommon but life-threatening clinical entity defined as splenic disruption occurring in the absence of antecedent trauma. Although it accounts for a small fraction of all splenic ruptures, reported mortality is higher than that of traumatic rupture, largely due to delayed recognition and rapid hemodynamic decompensation once bleeding occurs [1-4]. A broad range of underlying conditions has been associated with ASR, including acute and chronic infections, systemic inflammatory disorders, coagulopathies, pregnancy, and exposure to certain medications such as anticoagulants or thrombolytics [2-5]. Among these, hematologic malignancies represent one of the most frequent predisposing factors and are particularly important to consider when no clear alternative etiology is identified [3,4,6,7].

Hematologic cancers implicated in ASR include both lymphoid and myeloid neoplasms, most commonly non-Hodgkin lymphomas, acute and chronic leukemias, and less frequently myeloproliferative neoplasms [3-5,8]. In these settings, several pathophysiologic mechanisms may converge to weaken the splenic capsule and parenchyma, including diffuse neoplastic infiltration, vascular congestion, infarction, and microvascular thrombosis [3,4]. Progressive enlargement of the spleen increases intrasplenic pressure and stretches the capsule, predisposing it to spontaneous tearing even in the absence of trauma or significant physical exertion [3-5,9]. Because presenting symptoms, such as abdominal pain, dizziness, or hypotension, are nonspecific, a high index of suspicion is required to avoid diagnostic delay.

Splenic marginal zone lymphoma (SMZL) is a rare indolent B-cell non-Hodgkin lymphoma characterized by predominant splenic involvement, bone marrow infiltration, and frequent association with cytopenias [7,8]. Many patients are asymptomatic at diagnosis, and the disease is often detected incidentally on imaging or during evaluation of splenomegaly or cytopenias [7]. Despite its marked tropism for the spleen, ASR is an exceptionally uncommon complication of SMZL and has been described mainly in isolated case reports and small case series [5,6]. When rupture does occur, it may represent the first clinical manifestation of an otherwise unrecognized lymphoma, and the diagnosis is frequently established only after splenectomy [5-7].

Timely recognition of ASR is critical, as hemodynamic instability may develop rapidly and requires prompt resuscitation and urgent intervention. Contrast-enhanced computed tomography (CT) is the imaging modality of choice and can simultaneously demonstrate splenic lacerations, hemoperitoneum, and underlying splenomegaly or mass lesions [2-4,10]. Management strategies depend on hemodynamic status and institutional expertise, ranging from close observation and splenic artery embolization in selected stable patients to emergent splenectomy in those with ongoing hemorrhage or shock [1,3-5,10]. In the context of hematologic malignancy, splenectomy not only achieves definitive hemostasis but also provides tissue for accurate histopathologic and immunophenotypic characterization, which is essential for guiding subsequent oncologic management [6-8,10].

## Conclusions

Atraumatic splenic rupture is an uncommon but potentially fatal event that may serve as the initial presentation of an otherwise indolent hematologic malignancy such as splenic marginal zone lymphoma. Early recognition and prompt intervention are critical, as delayed diagnosis can lead to catastrophic hemorrhage and mortality. Splenectomy remains both diagnostic and therapeutic, allowing definitive histopathologic evaluation, stabilization, and long-term disease management. Clinicians should maintain a high index of suspicion for underlying hematologic malignancy in patients presenting with spontaneous splenic rupture, even in the absence of trauma or prior lymphoma diagnosis.
